# Precisely Printable Silk Fibroin/Carboxymethyl Cellulose/Alginate Bioink for 3D Printing

**DOI:** 10.3390/polym16081027

**Published:** 2024-04-09

**Authors:** Yuliya Nashchekina, Anastasia Militsina, Vladimir Elokhovskiy, Elena Ivan’kova, Alexey Nashchekin, Almaz Kamalov, Vladimir Yudin

**Affiliations:** 1 Institute of Cytology of the Russian Academy of Sciences, Center of Cell Technologies, St. Petersburg 194064, Russia; 2 Peter the Great St. Petersburg Polytechnic University, St. Petersburg 195251, Russia; nmnastya2103@gmail.com; 3 Institute of Macromolecular Compounds of Russian Academy of Sciences, St. Petersburg 199004, Russia; vladimir.elokhovskiy@gmail.com (V.E.); ivelen@mail.ru (E.I.); spb.kamalov@gmail.com (A.K.); 4 S.M. Kirov Military Medical Academy, Scientific Research Center, St. Petersburg 194044, Russia; 5 Ioffe Institute, Laboratory «Characterization of Materials and Structures of Solid State Electronics», St. Petersburg 194021, Russia; nashchekin@mail.ioffe.ru

**Keywords:** silk fibroin, carboxymethyl cellulose sodium, sodium alginate, 3D printing, mesenchymal stromal cells

## Abstract

Three-dimensional (3D) bioprinting opens up many possibilities for tissue engineering, thanks to its ability to create a three-dimensional environment for cells like an extracellular matrix. However, the use of natural polymers such as silk fibroin in 3D bioprinting faces obstacles such as having a limited printability due to the low viscosity of such solutions. This study addresses these gaps by developing highly viscous, stable, and biocompatible silk fibroin-based inks. The addition of 2% carboxymethyl cellulose sodium and 1% sodium alginate to an aqueous solution containing 2.5 to 5% silk fibroin significantly improves the printability, stability, and mechanical properties of the printed scaffolds. It has been demonstrated that the more silk fibroin there is in bioinks, the higher their printability. To stabilize silk fibroin scaffolds in an aqueous environment, the printed structures must be treated with methanol or ethanol, ensuring the transition from the silk fibroin’s amorphous phase to beta sheets. The developed bioinks that are based on silk fibroin, alginate, and carboxymethyl cellulose demonstrate an ease of printing and a high printing quality, and have a sufficiently good biocompatibility with respect to mesenchymal stromal cells. The printed scaffolds have satisfactory mechanical characteristics. The resulting 3D-printing bioink composition can be used to create tissue-like structures.

## 1. Introduction

Three-dimensional printing is a promising modern method of forming three-dimensional scaffolds for the restoration of damaged tissues and organs. The distinctive advantages of 3D printing technology in comparison with the classical methods of scaffold formation are its high automation/repeatability and precise control over the microstructures of the obtained scaffolds, as well as the possibility of creating an implant with a shape, which accurately reproduces the shape and size of each patient’s defect [1,2,3]. Biocompatible hydrogels are the most popular material for 3D-printed scaffolds, with the cells being designed to form tissue-like structures in vitro to allow for their further transplantation into a patient’s body [4,5]. Modern hydrogels used for 3D printing can be divided into hydrogels of synthetic and natural origin. Synthetic hydrogels include materials such as pluronics, polyethylene glycol, and poly-N-isopropylacrylamide [6,7]. Three-dimensional printing of scaffolds using hydrogels of synthetic origin makes it possible to control the microstructure of the obtained scaffolds with a level of high accuracy [8,9]. It should also be noted that such scaffolds have excellent mechanical properties. However, the degradation products of these scaffolds after their in vivo transplantation can have a toxic effect on the surrounding tissues. Therefore, despite having a number of advantages, the use of hydrogels of synthetic origin has significant disadvantages.

Three-dimensional printing with hydrogels obtained from polymers of natural origin allows biocompatible scaffolds to be formed. Scaffolds prepared on the basis of natural polymers such as collagen, chitosan, hyaluronic acid, etc., have demonstrated a high level of biocompatibility with respect to cells and tissues. The viability and proliferative activity of the cells cultured in these scaffolds, as a rule, indicate their biocompatibility [10,11]. The degradation products of the scaffolds after their transplantation into the body do not have a toxic effect on the surrounding tissues [12]. However, the printability of such hydrogels, as well as the mechanical properties of the printed scaffolds, have unsatisfactory indicators that need to be improved.

Hydrogels based on a natural polymer such as silk fibroin are an ideal material for tissue engineering. Silk fibroin (SF) has a low immunogenicity and good mechanical properties, as well as a high level of biocompatibility and it does not allow bacteria to adhere to it [13]. The possibility of obtaining an aqueous solution using silk fibroin would allow for its use in many areas of tissue engineering and regenerative medicine in the form of films, sponges, tubes, and gels [14]. For example, SF is used for the restoration of bone and cartilage tissues [15,16], and also for the restoration of soft tissues due to its mechanical properties [17,18]. However, the use of SF in the form of bioinks for 3D printing is limited by the low concentration of SF in the solution and its insufficient viscosity. A solution with a high concentration of SF can be obtained through dialysis in a solution of polyethylene glycol (PEG) with a high molecular weight (PEG, molecular weight (M_W_) 20,000 Da) followed by dissolution in an organic solvent [19,20].

However, the biological activity of SF proteins inevitably weakens as a result of these processes. There is also a method for the direct dissolution of SF in a mixture of organic solvents in the presence of formic acid to obtain a solution with an increased content of silk fibroin [21,22]. Nevertheless, the use of such solvents leads to the destruction of SF macromolecules and, as a consequence, to a decrease in both the molecular weight and the viscosity of the solution. In addition, organic solvents can have a toxic effect on living cells. When polymers are added to bioinks, it is not only their concentration that determines the print quality. The rapid transformation of a polymer solution into a gel is one of the most promising approaches. The SF produced in this way is biomorphic, and the SF solution can be spontaneously transformed from a liquid to a gel, thus failing 3D bioprinting [23]. The random helical amino acid sequence in SF can be converted into a β-sheet structure with excellent mechanical properties through inter- and intramolecular hydrogen bonding; a transformation that turns SF from a liquid into a gel [24]. Strong hydrogels based on SF can be prepared by exposing it to physical or chemical factors that ensure the formation of a water-insoluble β-phase, for example, photo-crosslinking, pH adjustment, or ultrasonication [25,26,27,28,29,30]. However, these methods are cytotoxic and can damage the cells seeded into the bioink. Although the β-sheet domains of the SF hydrogel provide stiffness and tensile strength, they exhibit poor elasticity [31]. In addition, these hydrogels are unlikely to be suitable for 3D printing since the grids within β-sheets can clog needles [3,32].

An alternative way to obtain high-viscosity SF solutions that are appropriate for 3D printing is to mix other high-viscosity polymers with SF, which is compatible with such biopolymers as gelatin, chitosan, alginate, and hyaluronic acid [33,34,35,36]. Cellulose and its derivatives are widely used in tissue engineering. Cellulose is a polysaccharide of natural origin, composed of linear chains of 1-4-linked β-d-anhydroglucopyranose units of variable length, generally synthesized by plants. Examples of the use of SF together with cellulose derivatives are known in the literature. For example, Huang and his co-authors developed inks based on silk fibroin and oxidized cellulose nanofibers [37]. SF backbones were cross-linked with horseradish peroxide (HRP)/H_2_O_2_ to form printed hydrogel scaffolds. Yan and co-authors added another cellulose derivative, hydroxypropyl cellulose, to SF to prepare an ink [23]. Methacrylate was compounded with hydroxypropyl cellulose to form hydroxypropyl cellulose methacrylate. Scaffolds can be formed using 3D printing based on such inks when exposed to UV radiation. However, UV radiation can have a negative effect on the viability of the cells inside gels. In our study, we propose a simple and affordable way to form bioinks for 3D printing. To increase the viscosity, we used carboxymethyl cellulose sodium (CMC).

CMC is the sodium salt of the carboxymethyl ether of cellulose, formed from the reaction of cellulose with monochloroacetic acid. CMC contains hydroxyl and carboxyl groups, which can produce rich hydrogen bonds in the hydrophobic region of SF to generate more β-sheet structures. SF and CMC are highly soluble in water; therefore, for their further use as bioinks, they must be stabilized and fixed. For this purpose, we utilized another natural, water-soluble biopolymer—alginate (Alg). Alginate is widely used as a bioink in the formation of scaffolds through 3D printing [38,39]. However, alginate has one important limitation—it has a negative charge. Therefore, the proliferative activity of the cells inside alginate gels is quite low. As a rule, bioinks which mainly include alginate are only applied for the cultivation of chondrocytes—cells of cartilage tissue that prefer to grow as aggregates.

The number of polymer materials that are suitable for the creation of bioinks is quite limited. In addition to the general requirements for materials utilized in tissue engineering tasks and regenerative medicine, these materials should only be soluble in aqueous solvents, the pH of which should be within the 5.5–7.5 range. Also, the viscosity values of the obtained solutions should be high enough to ensure the high quality of the printed structures obtained, as well as their stability over time. As an analysis of the literature data has shown, when mixing polymers with sufficiently high viscosity characteristics, it is not always possible to obtain bioinks that meet all of the requirements of bioprinting. Therefore, the development of an ink composition based on natural biocompatible materials that is suitable for high-quality printing in all respects is an important and relevant topic within modern tissue engineering.

The aim of this study Is to create a hydrogel with high printing parameters that does not clog the needles during printing, as well as to print biocompatible and mechanically strong scaffolds.

## 2. Materials and Methods

### 2.1. Preparation of SF Aqueous Solution

**Figure 1:**
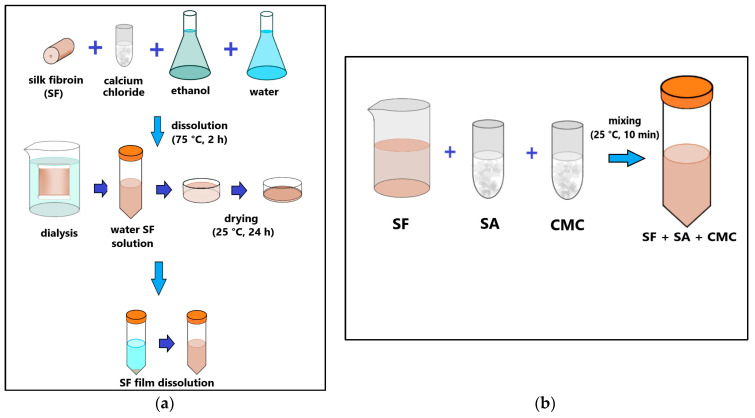
Scheme of SF solution (**a**) and bioink (**b**) preparation.

The aqueous solution of SF was prepared according to the method described in [14]. Surgical suture threads were used to obtain an aqueous SF solution (Mosnitki, Moscow, Russia). A diagram of the process is shown in Figure 1a. The filaments were dissolved in an aqueous mixture based on CaCl_2_ (Reagent, Moscow, Russia), C_2_H_5_OH (Reagent, Moscow, Russia), and H_2_O in a molar ratio of 1:2:8, respectively. The resulting mixture was kept at a temperature of 75 °C for 2 h in a water bath until the silk was completely dissolved. To remove calcium chloride, a dialysis solution was used against water. The water was changed 5 times during one day. The resulting supernatant was centrifuged. To obtain dry silk fibroin, the solution was poured onto Petri dishes and dried at room temperature until the water completely evaporated. Next, the dry silk fibroin was dissolved in distilled water to a final protein concentration of 2.5 or 5%.

A pure alginate and mixtures of alginate and carboxymethyl cellulose solutions with different concentration of silk fibroin (SF 2.5% and SF 5%) and alginate and CMC without silk fibroin (SF 0%) were prepared following the steps shown in Figure 1.

### 2.2. Preparation of Bioinks

To prepare bioinks, SF was dissolved in distilled water at room temperature. After the complete dissolution of SF (final concentration 2.5% (*w*/*v*) or 5% (*w*/*v*)), carboxymethyl cellulose sodium (Sigma-Aldrich, St. Louis, MO, USA) was added to the SF solution at a concentration of 2% (*w*/*v*) and alginate (Alg) (alginic acid sodium salt from brown algae (Sigma-Aldrich, St. Louis, MO, USA) at a concentration of 1% (*w*/*v*) with intensive stirring until all components were completely dissolved (Figure 1b). Thus, the bioink contained SF 2.5%, CMC, and alginate (sample SF 2.5%) or 5% SF, CMC, and alginate (sample SF 5%). The resulting bioinks were left at 4 °C for 2 days to remove air bubbles. The bioinks obtained by dissolving 2% CMC and 1% Alg in water without SF were used as a control (SF 0%).

After printing, the obtained scaffolds were fixed by treatment with a 2% solution of calcium chloride (Reagent, Moscow, Russia) for 5 min and methyl or ethyl alcohol for 10 min.

### 2.3. Rheology of Solutions

The rheological properties of the obtained bioinks were studied with the help of a rheometer MCR301 (Anton Paar, Stuttgart, Germany) in a CC17-SN8019970cylindrical measuring device. The tests were carried out in shear mode (at a constant rotation speed) with a step-by-step decrease in the deformation rate from 1000^−1^ to the minimum possible rate.

### 2.4. Printing

For the formation of volumetric scaffolds, the method of 3D extrusion printing was used with the help of a 3D bioprinter Dr. INVIVO (ROKIT Healthcare, Seoul, South Korea). By means of a pneumatic dispenser with a syringe, ink was supplied to a movable printing table. A 10 mL syringe with a 24G needle attachment was used for printing (24G needle nominal inner diameter = 0.311 mm, outer diameter = 0.566 mm according to Needle Gauge Chart).

The print model was created in the Autodesk Fusion 360 program and translated into a suitable G-code for the printer using NewCreatorK (version 1.57.71).

The following parameters were used for printing: printer type—Bio (print area width = 100 mm; print area depth = 100 mm; print area height = 90 mm) slicer type—Edislicer; nozzle size = 0.3 mm; print speed = 4 mm/s; input flow = 100%; printing temperature = 25 °C; infill pattern = lines.

The printer was controlled using a touch screen located on the top of the outside of the device; the pre-installed software was Android OS 7. The scaffolds obtained by 3D printing were treated with methyl or ethyl alcohol for 2 h to form an insoluble beta-folded structure of silk fibroin and 3% (*w*/*v*) calcium chloride solution for crosslinking sodium alginate. After precipitation, the scaffolds were thoroughly washed several times with distilled water.

### 2.5. IR-Fourier Spectroscopy

The molecular structure of the obtained scaffolds was evaluated with a IR Prestige-21 Fourier transform infrared spectrometer (FTIR) (Shimadzu, Tokyo, Japan) in the transmittance mode in the range of 4000–600 cm^−1^ with a resolution of 2 cm^−1^.

### 2.6. Print Quality

To determine the print quality, a 3D model of a grid with square cells with a given side length of 8 mm was used, according to which the matrices were printed. The suitability of formulations for 3D printing (*Pr*, from the English printability) was evaluated using the formula:

(1) $$Pr=\frac{L^{2}}{16\cdot A}$$

where *L* is the inner perimeter of the cell, and *A* is the area of the cell.

For a perfectly square cell, the printability is 1. If the ink has insufficient viscosity, the shape of the cells will be more rounded, and the *Pr* will decrease. FOr the same structures, the reduction in the pore area was estimated using the formula:

(2) $$D=\frac{S}{S_{0}}\cdot 100\%$$

where *S* is the actual area of the support, and *S*_0_ is the expected area.

The sagging of the thread was also evaluated according to the method proposed by Therriault and co-authors [40]. To do this, we used a comb with a variable distance between the teeth from 1 to 5 mm in increments of 1 mm. The studied gels were applied to its surface in the form of a continuous thread and the sagging factor (collapse factor) was determined using the formula:

(3) $$C=\frac{A}{A_{0}}\cdot 100\%$$

where *A* is the actual area under the gel thread, and *A*_0_ is the expected area.

The parameters (printability and collapse factor) were measured using the ImageJ program.

### 2.7. Dynamic Mechanical Analysis

Dynamic mechanical analysis of compression samples was carried out at the DMA 141 242 C installation (NETZSCH, Selb, Germany) at a constant temperature of 37 °C under a mechanical load of Fd = 0.5 N and with a frequency which varied from 0.1 to 100 Hz. The base height of the sample was 6 mm, and the diameter was 10 mm. The dependence of the elastic modulus E’(f) on the frequency of the load was constructed according to the experimental data obtained. Cubic spline interpolation was used to process the experimental curves.

### 2.8. Scanning Electron Microscopy

The microstructure of the scaffolds obtained by 3D printing was analyzed using a Supra-55VP scanning electron microscope (Carl Zeiss, Oberkochen, Germany). Before the scanning electron microscopy (SEM) investigation, the samples were glued onto special holders and sputtered with Pt.

### 2.9. Swelling

To assess the effect of silk fibroin content and the methods of beta-folding formation, the swelling of matrices in distilled water was studied. For this purpose, the matrices were dried in a thermostat at a temperature of 30 °C, weighed, and immersed in distilled water. The degree of swelling was calculated by the formula:

(4) $$\alpha (t)=\frac{m(t)}{m_{0}}$$

where *m*(*t*) is the mass of the matrix at time *t*, and *m*_0_ is the mass of the completely dried matrix.

To estimate the swelling rate, measurements were carried out after 1, 2, and 3 h, as well as 24 h hours after the beginning of swelling at room temperature.

### 2.10. In Vitro Studies

The printed scaffolds were dried until water was completely removed, sterilized under ozone exposure for 90 min, and then incubated in a complete DMEM/F12 nutrient medium (Dulbecco’s modified Eagle’s medium, Lonza, St. Louis, MO, USA) containing 10% (by volume) thermally inactivated fetal bovine serum (FBS; HyClone, St. Louis, MO, USA), 1% L-glutamine, 50 units/mL of penicillin, and 50 mcg/mL of streptomycin for 3 days. The resulting incubation medium was examined for cytotoxicity with respect to mesenchymal stromal cells.

Human FetMSCs cell line (human mesenchymal stromal cells—Institute of Cytology, St. Petersburg, Russia) was used to study cytotoxicity. Cells were cultured in a CO_2_ incubator at 37 °C in a humidified atmosphere containing air and 5% CO_2_ in a DMEM/F12 nutrient medium (Dulbecco’s modified Eagle’s medium; Lonza, St. Louis, MO, USA) containing 10% (by volume) thermally inactivated fetal bovine serum (FBS; HyClone, St. Louis, MO, USA), 1% L-glutamine, 50 units/mL of penicillin (Sigma-Aldrich, Steinheim, Germany), and 50 mcg/mL of streptomycin (Sigma-Aldrich, Steinheim, Germany).

For the experiment, 5 × 10^3^ cells/100 µL/well were sown in 96-well plates and cultured for 24 h for their attachment. A day later, the medium was drained and a complete nutrient medium was added to the wells after incubation with printed samples fixed with calcium chloride, in addition to ethanol or methanol for 3 days. At the end of the incubation period (3 days), the medium was removed and 50 µL/well of DMEM/F12 medium with MTT (3-(4,5-dimethylthiazole-2-yl)-2,5-diphenyl-tetrazolium bromide) (0.1 mg/mL) was added. The cells were incubated in a CO_2_ incubator for 2 h at 37 °C. After removal of the supraplastic fluid, formazane crystals formed by metabolically viable cells were dissolved in dimethyl sulfoxide (50 µL/well) and transferred to clean wells, and then the optical density was measured at 570 nm on a flatbed spectrophotometer.

### 2.11. Statistical Analysis

All experiments were repeated 5 times. A GraphPad Prism 8 two-way ANOVA and Tukey test were used to analyze the statistically significant differences between samples. Data were considered to be statistically important when *p* < 0.05.

## 3. Results and Discussion

SF consists of two main chains, a heavy (H-) chain and a light (L-) chain, which are linked via disulphide bonds to form an H-L complex [41,42]. P25 (25 KDa) is a glycoprotein which includes Asn-linked oligosaccharide chains and is hydrophobically linked to the H-L complex [43]. The H-chain, L-chain, and P25 are the three polypeptides that form a cocoon around B. mori and are found at a molar ratio of 6:6:1, respectively [44]. The amino acid sequence of the H-chain consists of Glycine (45.9%), Alanine (30.3%), Serine (5.3%), and Valine (1.8%), as well as 4.5% of 15 other types of amino acids [45].

Alginate is composed of (1-4)-linked β-Dmannuronic (M) and α-Lguluronic acids (G). This material is a negatively charged linear copolymer (M and G blocks), which is soluble in water. The G-block of this material assists in the formation of gels and GM and M blocks by improving their flexibility.

Carboxymethyl cellulose is an anionic water-soluble biopolymer derived naturally or through a chemical reaction with cellulose. It is a copolymer of β-D-glucose and β-D-glucopyranose-2-O-(carboxymethyl)-monosodium salt, which are connected via β-1,4-glucosidic bonds [46].

SF, sodium alginate, and CMC are polar solutes which are soluble in water. These three materials carry out intermolecular action through the formation of hydrogen bonds and, consequently, can be used to produce compatible, blended, hybrid hydrogels [43,44,45].

### 3.1. Rheological Properties

Among the other types of printing, extrusion-based bioprinting has attracted a lot of attention due to its ability to use a wide range of materials and compositions [47].

Printability in 3D extrusion printing is defined as (1) the extrudability of bio-ink during extrusion, characterized by rheological properties, and (2) the formability of filaments after extrusion, characterized by the accuracy of the shape of filaments and structures. The rate of gelation can be controlled by adjusting the rheological properties of hydrogels by adding a viscosity modifier [48], or an external crosslinking agent [49], or through temperature changes [50,51]. However, improving viscosity does not always enhance the biocompatibility aspect of a bioink. For example, increasing viscosity will improve print quality and shape accuracy, but when increasing the viscosity of bioinks, it is also necessary to increase the extrusion pressure, which is harmful to the living cells contained in the bioink.

The rheological properties of bioinks play a crucial role in 3D printing processes. The aim of this experiment was to analyze the rheological differences from different silk fibroin concentrations. When the hydrogel is extruded under pressure through a small nozzle, it experiences shear stress. It should be in a solution state as it passes through the nozzle, but once extruded, it should retain the printed shape.

**Figure 2:**
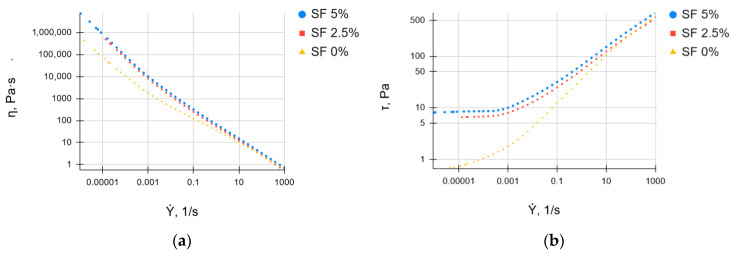
Dependences of viscosity (**a**) and shear stress (**b**) of the studied bioink on the shear rate.

Figure 2 shows the dependence of the effective viscosity and shear stress of the studied solutions based on SF on the shear rate. With an increase in the shear rate, the viscosity of all gels decreases (Figure 2a), indicating the destruction of the original structure and the formation of a new structure due to the orientation of polymer molecules along the shear field. At the same time, the shear stress increases as the shear rate increases (Figure 2b). An investigation of the rheological properties, which are crucial for determining the suitability of hydrogels for 3D printing, has shown that SF-based hydrogels are able to shift [52,53]. This means that the viscosity of the hydrogel decreases with the increasing shear rate.

Bioinks can be characterized as plastic and thixotropic at all of the studied concentrations of SF. As the proportion of SF in the ink increases, the viscosity of the solutions increases, that allows the gel to retain its shape during printing. As result, this is a prerequisite for using the gel as an ink in 3D bioprinting [54,55].

The overall cell viability of scaffolds manufactured by 3D bioprinting based on extrusion depends on the shear stress experienced by the encapsulated cells. There are data in the literature comparing cell viability and print quality based on the rheological characteristics of bioinks. The creation of scaffolds with a higher percentage of solids content can provide better shape accuracy [56], as this increases the viscosity of the composition. However, due to the higher dosing pressure required, the small nozzle diameter, and the corresponding higher shear stress, this can adversely affect the overall viability of the cells.

Based on the analysis of the literature data [57] and our own research, we can assume that the rheological properties obtained for the bioinks we developed will lead to a high print quality and a sufficiently high viability of the cells cultured inside the printed scaffolds.

### 3.2. Printability

**Figure 3:**
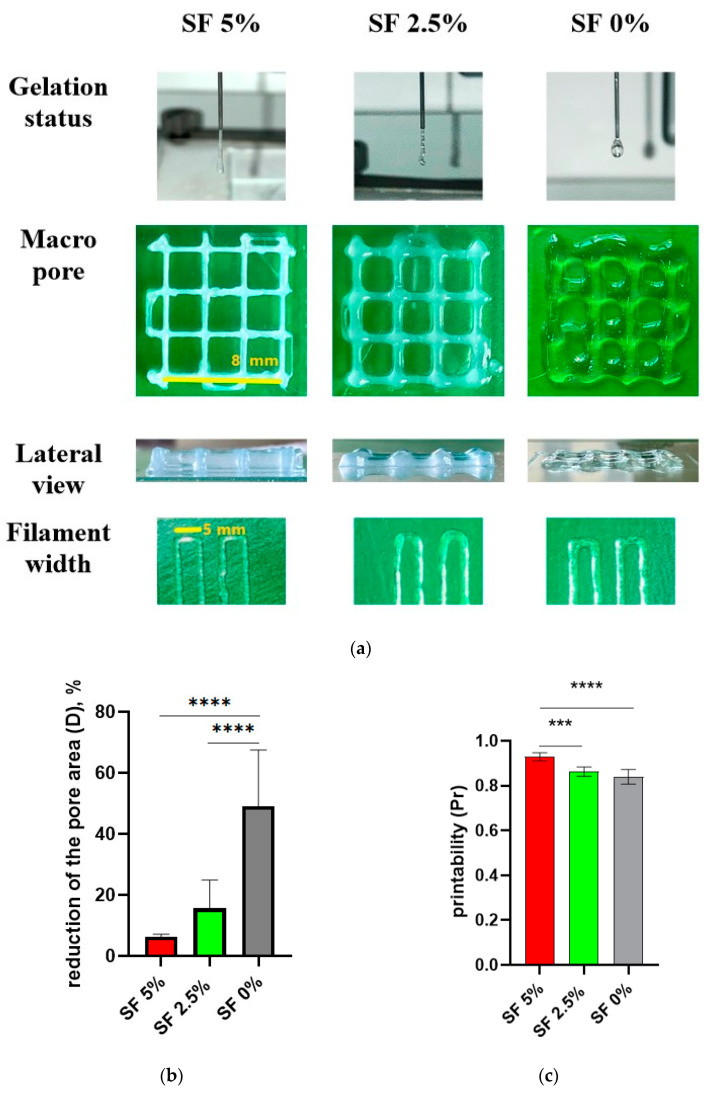
Filament fusion test (**a**). Reduction in the pore area (**b**) and printability (**c**) in the filament fusion test. *** *p* ≤ 0.001, **** *p* ≤ 0.0001.

The design of the cubic frame was chosen as a model system for determining the optimal composition of the hydrogel to achieve a high morphological accuracy in 3D printing (Figure 3a). These optimal component ratios can be used to print larger and more complex scaffolds. In our study, we aimed to develop hydrogels that were optimized for 3D printing using SF-based formulations with a well-defined gelation time, making them suitable for application in regenerative medicine. The size of the objects prepared with the help of the 3D printer was measured using a piece of software, and a comparison was made between the dimensions of the designed 3D objects and the actual printing result (Figure 3b). The measurements show a close similarity between the modeling and the results, especially in the group containing SF 5%. In the groups with a lower concentration of SF or without SF, it has been demonstrated that the filament diameter of printed products is larger, while the height is smaller than the design’s dimensions. This phenomenon can be explained by the fact that the ink is squeezed out of the nozzle and spreads slightly to the sides before being fixed with calcium chloride or alcohol.

Previously, in the literature, an ink based on CMC and alginate was presented [58]. The authors managed to significantly improve the print quality by increasing the concentrations of alginate and CMC in the hydrogel. Of course, the viscosity of such inks increased significantly, but a dense mesh of polymers and a negatively charged alginate would negatively affect the spreading and migration of cells inside such hydrogels. SF is known to consist mainly of amino acids such as alanine (Ala), glycine (Gly), serine (Ser), and others. These amino acids form hydrogen bonds within the silk molecule that are a crucial factor determining the strength and stability of the silk. Hydrogen bonds can take various forms within the silk molecule, and among them -OH (hydroxyl) groups play a significant role in the formation of hydrogen bonds [59].

**Figure 4:**
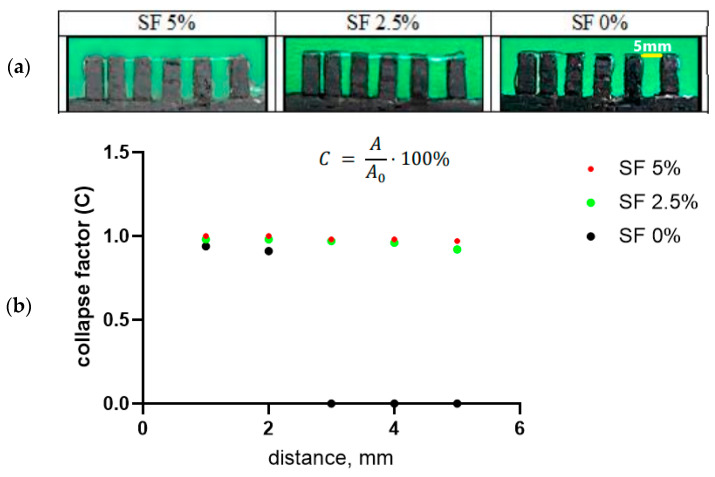
Filament collapse test (**a**) and collapse area factor (**b**).

The collapse factor of the gel thread makes it possible to assess the stability of a separately applied strip of composition against sagging in the air (Figure 4a). To do this, one strip of gel is applied to a comb with a variable distance between the teeth (from 1 to 5 mm, step 1 mm) using a printer. In this case, the printer provides the same application conditions—the volume of ink supplied, the speed of movement of the syringe, the pressure on the piston, and the distance between the syringe and the comb. In this case, the gel was applied at a syringe movement speed of 4 mm/s at an input flow value of 100%. With sufficient viscosity, the thread sags minimally; such a composition ensures a satisfactory print quality (Figure 4b). A high level of subsidence (up to the interruption of the thread) indicates that the ink viscosity is too low.

Thus, a gel with a 5% content of SF has the most satisfactory print quality indicators, which allows us to conclude that this composition is optimal for use as a bioink. The suitability for printing and the collapse factor of this composition are close to one, and the reduction in the cell area of the printed structure is minimal among the three studied gels. At the same time, there is a tendency for the print quality indicators to deteriorate with a decrease in the proportion of silk fibroin in the ink composition.

### 3.3. FTIR-Spectroscopy

**Figure 5:**
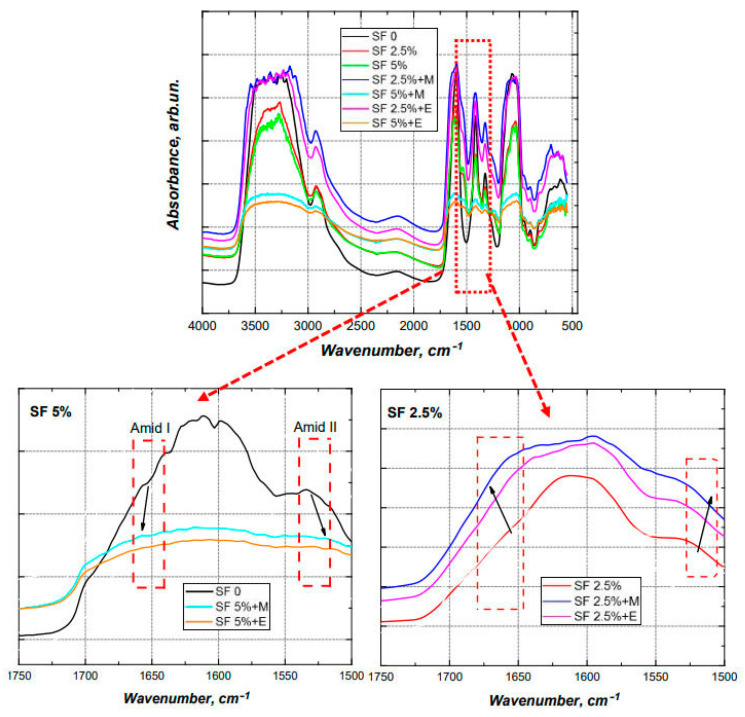
FTIR spectra of the printed scaffolds. SF 0%—printed scaffolds prepared using CMC, and sodium alginate; SF 2.5%—printed scaffolds prepared using CMC, sodium alginate, and 2.5% SF; SF 5%—printed scaffolds prepared using CMC, sodium alginate, and 5% SF; SF 2.5% + E—printed scaffolds prepared using CMC, sodium alginate, and 2.5% SF with treated ethanol; SF 5% + E—printed scaffolds prepared using CMC, sodium alginate, and 5% SF treated with ethanol; SF 2.5% + M—printed scaffolds prepared using CMC, sodium alginate, and 2.5% SF treated with methanol; SF 5% + M—printed scaffolds prepared using CMC, sodium alginate, and 5% SF treated with methanol.

In order to induce the formation of a crystalline β-structure, the scaffolds were treated with alcohols. In the present study, the effect of methyl and ethyl alcohols on the conformational transition of silk fibroin was compared (Figure 5). It is well known that, when silk fibroin is treated with polar alcohol, a conformational transition of the silk fibroin solution from a random spiral crystallization to β-sheets crystallization occurs [59]. As a result of this crystallization, physical cross-links are formed in the silk structure, which ensure the insolubility of the silk fibroin in water. Um and co-authors proposed a crystallization mechanism [60]. According to this mechanism, polar groups of alcohols attract water from silk fibroin molecules leading to an increased aggregation of hydrophobic amino acids, particularly Gly and Ala, in the internal structure of the silk fibroin molecules. The polarity of the alcohol was an important factor that regulated the transition of the silk fibroin from a random spiral crystallization to β-sheets. It was also previously reported that, as the length of the carbon chain increased, the polarity of the alcohol decreased, which led to an increased hydrophobic interaction between the alcohol and the silk fibroin molecules [23]. Consequently, ethanol can contribute to a more rapid crystallization of the β-layer of silk fibroin compared with methanol.

Changes in the structure of the scaffolds were investigated using Fourier transform IR spectroscopy on samples with different compositions before and after treatment with alcohols (Figure 5) [61].

The characteristic absorption peaks of sodium alginate can be seen at 1150 cm^−1^ (valence vibrations of the C-OH fragment), 1100 cm^−1^, 1420 cm^−1^ (valence symmetric vibrations of the polyanion carboxyl group), 1590–1610 cm^−1^ (valence antisymmetric vibrations of the polyanion carboxyl group), and 2890–2920 cm^−1^, and those of CMC can be seen at 1327 cm^−1^ (deformation vibrations CH_2_), 1610–1620 cm^−1^ (valence vibrations carboxyl group), 2914–2939 cm^−1^, and 3427 cm^−1^ [62,63,64].

Characteristic signals indicating the formation of a β-sheets structure were detected, including the band at 3070–3600 cm^−1^ (fluctuations of N-H and O-H peptide groups), the sharp drop in absorption by 1700 cm^−1^, the expansion of the band at 1590–1620 cm^−1^ to the boundaries of 1560–1650 cm^−1^, the fact that 1268 cm^−1^ shifted by 1230 cm^−1^ (amid III), the higher absorption at the interval 1070–1150 cm^−1^, a shift of 1540 cm^−1^ by 1518 cm^−1^ (amid II), and the shift of 1655 cm^−1^ to 1670–1674 cm^−1^ (amid I) [65].

The spectra of the films with the same content of silk fibroin after the conformational transition, regardless of which alcohol they were treated with, coincide with each other, which confirms the conformational transition of silk fibroin when interacting with both alcohols.

### 3.4. Swelling

SF dissolves well in aqueous solutions. Therefore, for further use of the printed scaffolds in vitro or in vivo, it is necessary to transform it into an insoluble form. An additional advantage of SF is the simplicity of its conversion into an insoluble form. When SF is treated with solutions of ethyl or methyl alcohol, SF is conformationally converted into β-sheets that are insoluble in water [65]. In our work, we studied the effect of ethanol and methanol on the ability of these alcohols to facilitate the conformational transition of SF in the presence of CMC and alginate.

**Figure 6:**
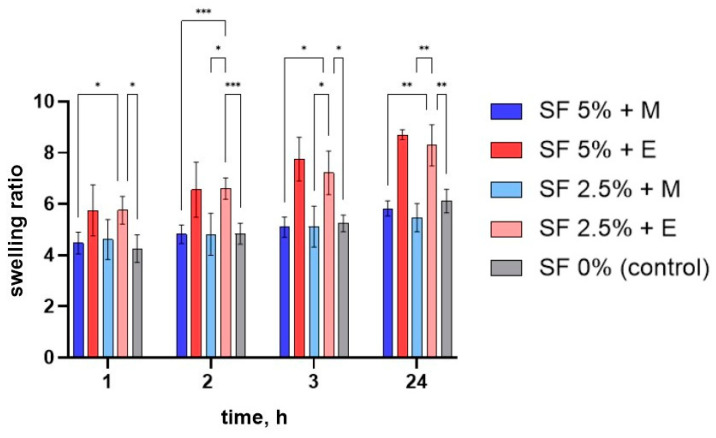
Swelling of the samples over time. SF 0%—printed scaffolds prepared using CMC and sodium alginate; SF 2.5% + E—printed scaffolds prepared using CMC, sodium alginate, and 2.5% SF treated with ethanol; SF 5% + E—printed scaffolds prepared using CMC, sodium alginate, and 5% SF treated with ethanol; SF 2.5% + M—printed scaffolds prepared using CMC, sodium alginate, and 2.5% SF treated with methanol; SF 5% + M—printed scaffolds prepared using CMC, sodium alginate, and 5% SF treated with methanol. * *p* ≤ 0.05, ** *p* ≤ 0.01, *** *p* ≤ 0.001.

To assess the swelling of printed scaffolds made by different methods, their degree of swelling was determined. For this purpose, the printed scaffolds were dried at room temperature for 48 h, weighed, and then immersed in water and the change in mass was tracked over time. Measurements were made 1, 2, 3, and 24 h after the beginning of swelling. After a day, all of the studied scaffolds were found to have swelled as much as possible (after 24 h they no longer absorbed water). The dynamics of the swelling of the studied scaffolds are shown in Figure 6.

The greatest degree of swelling was noted for matrices made of a gel with a 5% content of silk fibroin treated with ethyl alcohol—fully swollen samples are, on average, 8.7 times heavier than those in dried form. In the presence of alcohols, silk fibroin changes from its amorphous hydrophilic form to a crystalline hydrophobic one. As a result, it becomes insoluble in water, and its ability to absorb water is also reduced. This may indicate that the proportion of β-sheet structure formed after the SF is treated with ethanol is lower than when it interacts with methanol.

The samples had a lower degree of swelling at all stages after the methanol treatment compared to that after the ethanol treatment. Ethanol has a lower polarity O-H bond, so reactions associated with the destruction of this bond proceed more slowly. During the interaction of alcohols with amorphous silk fibroin, polar groups of alcohols attract water from silk fibroin molecules, leading to an increased aggregation of hydrophobic amino acids (especially glycine and alanine), which are part of protein molecules. Thus, by using alcohols of different polarities, it is possible to regulate the formation of beta structures, and, hence, the ability of the product to swell [59].

### 3.5. Dynamic Mechanical Analysis

**Figure 7:**
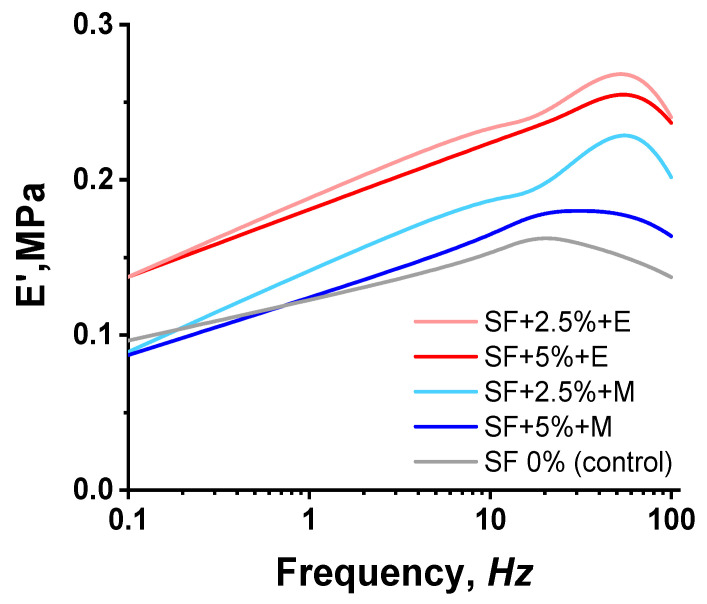
Dynamic mechanical analysis. SF 0%—printed scaffolds prepared using CMC and sodium alginate; SF 2.5% + E—printed scaffolds prepared using CMC, sodium alginate, and 2.5% SF treated with ethanol; SF 5% + E—printed scaffolds prepared using CMC, sodium alginate, and 5% SF treated with ethanol; SF 2.5% + M—printed scaffolds prepared using CMC, sodium alginate, and 2.5% SF treated with methanol; SF 5% + M—printed scaffolds prepared using CMC, sodium alginate, and 5% SF treated with methanol.

The DMA data demonstrate an increase in the rigidity of printed structures when SF was added (Figure 7). A higher SF content of 5% does not provide an increase in stiffness compared to a sample containing 2.5% SF. However, it is noted that the treatment of printed scaffolds with ethanol makes it possible to obtain more rigid scaffolds compared to scaffolds treated with methanol.

As previously noted, ethanol should contribute to the most rapid crystallization of the β-sheets of silk fibroin compared to methanol due to the longer length of its carbon chains, and, consequently, there should be an increased number of hydrophobic interactions between the alcohol and the silk fibroin molecules. In our study, it has been shown that, when treating the scaffold with ethanol, which has a longer carbon chain length compared to methanol, the elasticity of the scaffold decreases. This unexplained connection was also obtained in the work of Kaewpiroma and Boonsang [59].

It would be more logical to expect that, with an increase in the concentration of SF in the composition of bioinks, the modulus would increase. However, we observe an inverse relationship. We explain the results obtained by the fact that with an increase in the concentration of SF, its ability to mix evenly with other components of bioinks, namely alginate and CMC, decreases. SF macromolecules, as well as sodium alginate and CMC, have a sufficiently large size, so it is quite difficult to obtain a uniform solution with an increase in the concentration of polymers. Naturally, with an increase in the concentration of SF, defects can form that will affect the modulus value.

When 2.5% SF is added to the bioinks, a more uniform distribution occurs and a grid structure may be created, providing a high modulus of elasticity. To clarify this assumption, the structure of the samples was studied using SEM.

### 3.6. Scanning Electron Microscopy

**Figure 8:**
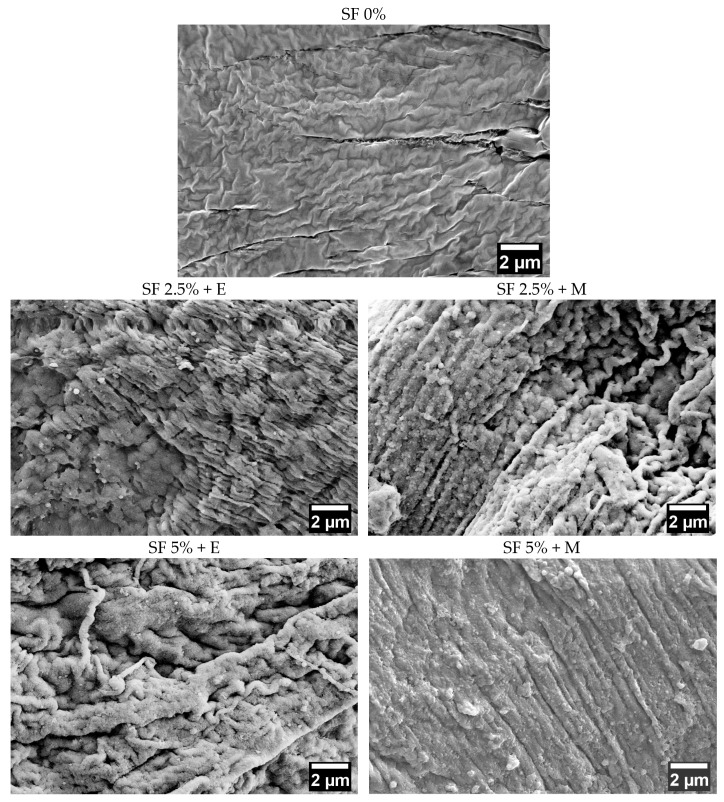
Scanning electron microscopy images. SF 0%—printed scaffolds prepared using CMC and sodium alginate; SF 2.5% + E—printed scaffolds prepared using CMC, sodium alginate, and 2.5% SF treated with ethanol; SF 5% + E—printed scaffolds prepared using CMC, sodium alginate, and 5% SF treated with ethanol; SF 2.5% + M—printed scaffolds prepared using CMC, sodium alginate, and 2.5%-SF treated methanol; SF 5% + M—printed scaffolds prepared using CMC, sodium alginate, and 5% SF treated with methanol.

The structure of the studied samples was investigated using the SEM technique. Figure 8 shows the SEM images of the scaffolds which differ in their silk fibroin content and the method of inducing the silk fibroin’s conformational transition. There are significant differences between samples that do not contain SF and the rest: for all scaffolds with SF, the formation of an oriented parallel structure corresponding to the β-sheets form of SF is revealed. The control sample, made only of CMC and sodium alginate, does not have such a structure.

Controlling the alignment and orientation of the scaffold surface was earlier reported to represent a wide range of applications, including improving the structural properties of scaffolds in addition to enhancing the growth of cells in nanofiber scaffolds for tissue engineering [66,67]. Thus, aligning silk fibroin nanofibers along their longitudinal direction may also have promising applications in tissue engineering by mimicking natural tissues or organs.

### 3.7. Cytotoxicity Test

**Figure 9:**
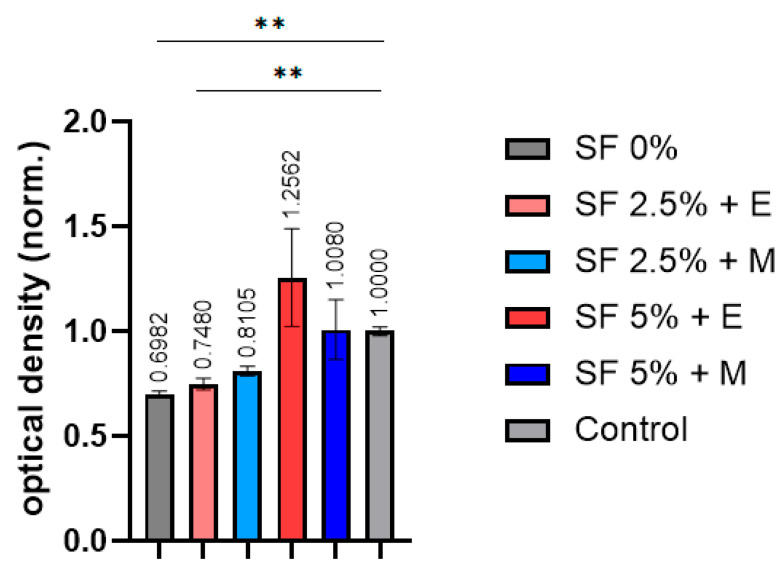
MTT test results. SF 0%—printed scaffolds prepared using CMC and sodium alginate; SF 2.5% + E—printed scaffolds prepared using CMC, sodium alginate, and 2.5% SF treated with ethanol; SF 5% + E—printed scaffolds prepared using CMC, sodium alginate, and 5% SF treated with ethanol; SF 2.5% + M—printed scaffolds prepared using CMC, sodium alginate, and 2.5% SF treated with methanol; SF 5% + M—printed scaffolds prepared using CMC, sodium alginate, and 5% SF treated with methanol. (** *p* ≤ 0.01).

To obtain functional and tissue-like structures, it is important to take into account the various stages of development that the printed scaffolds must go through. Cell viability is the very first step in evaluating the suitability of a scaffold for cell culture. Silk-based bioinks are known to play a key role in supporting the proliferation of cells within the printed construct [68]. Its degradation rate can be precisely controlled, varying from weeks to years following implantation in vivo, which in turn depends on the scaffold type, the cross-linking agent, crystallization, and other factors involved. It follows from the MTT test results that an increase in the proportion of SF contributes to an increase in the biocompatibility of the scaffolds (Figure 9).

In the samples printed without the addition of silk fibroin, the cell viability was found to be significantly lower than expected. The scaffolds with 2.5% silk fibroin provided more viable MSCs compared to the samples without SF, but this was still lower than expected. The scaffolds printed from bioinks with 5% silk fibroin provided a cell viability that was closer to what we expected. At the same time, the choice of alcohol does not have a negative effect on cell viability. The toxic effect of methanol on cells is known. The absence of a decrease in the viability of cells cultured in the medium after the incubation of the printed SF scaffolds in a nutrient medium indicates that methanol is only involved in the crystallization of silk fibroin into β-sheets and is not absorbed into the scaffolds. It is possible to say that, qualitatively, SF scaffolds offer a significant potential to be used in applications where cytocompatibility is required.

Indeed, significant differences compared with the control were only observed in samples based on CMC and alginate, as well as a sample containing CMC, alginate, and 2.5% silk fibroin. The decrease in cell viability in these samples is apparently due to the partial solubility of the components of these bioinks in the nutrient medium, which reduces the viability of cells cultured in such an incubation medium.

## 4. Conclusions

Over the course of this study, a new biocompatible hybrid hydrogel based on SF, CMC, and sodium alginate has been developed and optimized for use in 3D printing. To check its suitability for printing and the accuracy of the printed form, a number of systematic quantitative tests have been carried out. The results of the rheological and dynamical mechanical analyses demonstrate a favorable shape accuracy. These performance tests show that the composition of 5% SF, 2% CMC, and 1% alginate provides a good printability and shape accuracy for large-scale frame manufacturing. For the manufacture of large-scale functional tissue frameworks, this hybrid hydrogel can become a potential biomaterial for the process of 3D bioprinting, and the methods described for determining the scaffolds’ characteristics pave the way to ensure the reproducibility of printing and the accuracy of the printed form. The future tasks that should be carried out in this research are the creation of large-scale tissue scaffolds saturated with cells, with various types of functional cells, and determining the physiological behavior of a joint culture in a cells’ microenvironment.

## Data Availability

Data available upon request.

## References

[1] Donderwinkel I., Hest J.C.M.V., Cameron N.R. (2017). Bio-inks for 3D bioprinting: Recent advances and future prospects. Polym. Chem..

[2] Costa J.B., Silva-Correia J., Oliveira J.M., Reis R.L. (2017). Fast setting silk fibroin bioink for bioprinting of patient-specicific memory-shape implants. Adv. Healthc. Mater..

[3] Wodarczyk-Biegun M.K., del Campo A.D. (2017). 3D bioprinting of structural proteins. Biomaterials.

[4] Hospodiuk M., Dey M., Sosnoski D., Ozbolat I.T. (2017). The bioink: A comprehensive review on bioprintable materials. Biotechnol. Adv..

[5] Li J., Wuc C., Chub P.K., Gelinskya M. (2020). 3D printing of hydrogels: Rational design strategies and emerging biomedical applications. Mat. Sci. Eng. R Rep..

[6] Kim J.E., Kim S.H., Jung Y. (2016). Current status of threedimensional printing inks for soft tissue regeneration. Tissue Eng. Regener. Med..

[7] Kaliaraj G.S., Shanmugam D.K., Dasan A., Mosas K.K.A. (2023). Hydrogels—A Promising Materials for 3D Printing Technology. Gels.

[8] Arguchinskaya N.V., Isaeva E.V., Kisel A.A., Beketov E.E., Lagoda T.S., Baranovskii D.S., Yakovleva N.D., Demyashkin G.A., Komarova L.N., Astakhina S.O. (2023). Properties and Printability of the Synthesized Hydrogel Based on GelMA. Int. J. Mol. Sci..

[9] Hu C., Hahn L., Yang M., Altmann A., Stahlhut P., Groll J., Luxenhofer R. (2021). Improving printability of a thermoresponsive hydrogel biomaterial ink by nanoclay addition. J. Mater. Sci..

[10] Xie F. (2023). Chapter 4—3D Printing of Biopolymer-Based Hydrogels. Additive Manufacturing of Biopolymers. Handbook of Materials, Techniques, and Applications.

[11] Ramiah P., du Toit L.C., Choonar Y.E., Kondiah P.P.D., Pillay V. (2020). Hydrogel-Based Bioinks for 3D Bioprinting in Tissue Regeneration. Front. Mater..

[12] Hama R., Ulziibayar A., Reinhardt J.W., Watanabe T., Kelly J., Shinoka T. (2023). Recent Developments in Biopolymer-Based Hydrogels for Tissue Engineering Applications. Biomolecules.

[13] Kambe Y. (2021). Functionalization of silk fibroin-based biomaterials for tissue engineering. Polym. J..

[14] Nashchekina Y.A., Konygina V.S., Popova E.N., Kodolova-Chukhontseva V.V., Nashchekin A.V., Yudin V.E. (2022). Preparation of water-insoluble silk fibroin films. study of their structure and properties. Tech. Phys..

[15] Rockwood D.N., Gil E., Park S.-H., Kluge J., Grayson W., Bhumiratana S., Rajkhowa R., Wang X., Kim S., Vunjak-Novakovic G. (2011). Ingrowth of human mesenchymal stem cells into porous silk particle reinforced silk composite scaffolds: An in vitro study. Acta Biomater..

[16] Baek H.S., Park Y., Ki C., Park J.-C., Rah D.K. (2008). Enhanced chondrogenic responses of articular chondrocytes onto porous silk fibroin scaffolds treated with microwave-induced argon plasma. Surf. Coat. Technol..

[17] Chen X., Qi Y.Y., Wang L.L., Yin Z., Yin G.L., Zou X.H., Ouyang H.W. (2008). Ligament regeneration using a knitted silk scaffold combined with collagen matrix. Biomaterials.

[18] Sun K., Li H., Li R., Nian Z., Li D., Xu C. (2015). Silk fibroin/collagen and silk fibroin/chitosan blended three-dimensional scaffolds for tissue engineering. Eur. J. Orthop. Surg. Traumatol..

[19] Yao D., Dong S., Lu Q., Hu X., Kaplan D.L., Zhang B., Zhu H. (2012). Salt-leached silk scaffolds with tunable mechanical properties. Biomacromolecules.

[20] Zhu Z.H., Ohgo K., Asakura T. (2008). Preparation and characterization of regenerated *Bombyx mori* silk fibroin fiber with high strength. Express Polym. Lett..

[21] Ling S., Zhang Q., Kaplan D.L., Omenetto F., Buehler M.J., Qin Z. (2016). Printing of stretchable silk membranes for strain measurements. Lab Chip.

[22] Zhang F., You X., Dou H., Liu Z., Zuo B., Zhang X. (2015). Facile fabrication of robust silk nanofibril films via direct dissolution of silk in CaCl2-formic acid solution. ACS Appl. Mater. Interfaces.

[23] Yan K., Zhang X., Liu Y., Cheng J., Zhai C., Shen K., Liang W., Fan W. (2023). 3D-bioprinted silk fibroin-hydroxypropyl cellulose methacrylate porous scaffold with optimized performance for repairing articular cartilage defects. Mater. Des..

[24] Kaewprasit K., Kobayashi T., Damrongsakkul S. (2018). Thai silk fibroin gelation process enhancing by monohydric and polyhydric alcohols. Int. J. Biol. Macromol..

[25] Su D.H., Yao M., Liu J., Zhong Y., Chen X., Shao Z.Z. (2017). Enhancing mechanical properties of silk fibroin hydrogel through restricting the growth of b-sheet domains. ACS Appl. Mater. Interfaces.

[26] Luo K.Y., Yang Y.H., Shao Z.Z. (2016). Physically crosslinked biocompatible silk-fibroin-based hydrogels with high mechanical performance. Adv. Funct. Mater..

[27] Vuornos K., Björninen M., Talvitie E., Paakinaho K., Kellomäki M., Huhtala H., Miettinen S., Seppänen-Kaijansinkko R., Haimi S. (2016). Human Adipose Stem Cells Differentiated on Braided Polylactide Scaffolds Is a Potential Approach for Tendon. Tissue Eng. Part A.

[28] Dodel M., Hemmati Nejad N., Bahrami S.H., Soleimani M., Mohammadi Amirabad L., Hanaee-Ahvaz H., Atashi A. (2017). Electrical stimulation of somatic human stem cells mediated by composite containing conductive nanofibers for ligament regeneration. Biologicals.

[29] Font Tellado S., Chiera S., Bonani W., Poh P.S.P., Migliaresi C., Motta A., Balmayor E.R., van Griensven M. (2018). Heparin functionalization increases retention of TGF-b2 and GDF5 on biphasic silk fibroin scaffolds for tendon/ligament-tobone tissue engineering. Acta Biomater..

[30] Yuan T., Li Z., Zhang Y., Shen K., Zhang X., Xie R., Liu F., Fan W. (2021). Injectable Ultrasonication-Induced Silk Fibroin Hydrogel for Cartilage Repair and Regeneration. Tissue Eng. Part A.

[31] Silva-Correia J., Ribeiro V.P., Miranda-Gonçalves V., Correia C., da Silva Morais A., Sousa R.A., Reis R.M., Oliveira A.L., Oliveira J.M., Reis R.L. (2016). Tumor growth suppression induced by biomimetic silk fibroin hydrogels. Sci. Rep..

[32] Desimone E., Schacht K., Jungst T., Groll J., Scheibel T. (2015). Biofabrication of 3D constructs: Fabrication technologies and spider silk proteins as bioinks. Pure Appl. Chem..

[33] Wang Q., Han G., Yan S., Zhang Q. (2019). 3D Printing of Silk Fibroin for Biomedical Applications. Materials.

[34] Jose R.R., Brown J.E., Polido K.E., Omenetto F.G., Kaplan D.L. (2015). Polyol-Silk Bioink Formulations as Two-Part Room-Temperature Curable Materials for 3D Printing. ACS Biomater. Sci. Eng..

[35] Kim S.H., Yeon Y.K., Lee J.M., Chao J.R., Lee Y.J., Seo Y.B., Sultan M.T., Lee O.J., Lee J.S., Yoon S.I. (2018). Precisely printable and biocompatible silk fibroin bioink for digital light processing 3D printing. Nat. Commun..

[36] Chameettachal S., Midha S., Ghosh S. (2016). Regulation of Chondrogenesis and Hypertrophy in Silk Fibroin-Gelatin-Based 3D Bioprinted Constructs. ACS Biomater. Sci. Eng..

[37] Huang L., Yuan W., Hong Y., Fan S., Yao X., Ren T., Song L., Yang G., Zhang Y. (2021). 3D printed hydrogels with oxidized cellulose nanofibers and silk fibroin for the proliferation of lung epithelial stem cells. Cellulose.

[38] Axpe E., Oyen M.L. (2016). Applications of Alginate-Based Bioinks in 3D Bioprinting. Int. J. Mol. Sci..

[39] Datta S. (2023). Advantage of Alginate Bioinks in Biofabrication for Various Tissue Engineering Applications. Int. J. Polym. Sci..

[40] Therriault D., White S.R., Lewis J.A. (2007). Rheological behavior of fugitive organic inks for direct-write assembly. Appl. Rheol..

[41] Qi Y., Wang H., Wei K., Yang Y., Zheng R.-Y., Kim I.S., Zhang K.-Q. (2017). A Review of Structure Construction of Silk Fibroin Biomaterials from Single Structures to Multi-Level Structures. Int. J. Mol. Sci..

[42] Tomeh M.A., Hadianamrei R., Zhao X. (2019). Silk Fibroin as a Functional Biomaterial for Drug and Gene Delivery. Pharmaceutics.

[43] Tanaka K., Inoue S., Mizuno S. (1999). Hydrophobic interaction of P25, containing Asn-linked oligosaccharide chains, with the H-L complex of silk fibroin produced by *Bombyx mori*. Insect Biochem. Mol. Biol..

[44] Inoue S., Tanaka K., Arisaka F., Kimura S., Ohtomo K., Mizuno S. (2000). Silk fibroin of *Bombyx mori* is secreted, assembling a high molecular mass elementary unit consisting of H-chain, L-chain, and P25, with a 6:6:1 molar ratio. J. Biol. Chem..

[45] Sun W., Gregory D.A., Tomeh M.A., Zhao X. (2021). Silk Fibroin as a Functional Biomaterial for Tissue Engineering. Int. J. Mol. Sci..

[46] Han Y., Wang L. (2017). Sodium alginate/carboxymethyl cellulose films containing pyrogallic acid: Physical and antibacterial properties. J. Sci. Food Agric..

[47] Wang L.L., Highley C.B., Yeh Y.C., Galarraga J.H., Uman S., Burdick J.A. (2018). Three-dimensional extrusion bioprinting of single- and double-network hydrogels containing dynamic covalent crosslinks. J. Biomed. Mater. Res. A.

[48] Ahlfeld T., Köhler T., Czichy C., Lode A., Gelinsky M. (2018). A Methylcellulose Hydrogel as Support for 3D Plotting of Complex Shaped Calcium Phosphate Scaffolds. Gels.

[49] Kuo C., Qin H., Favio Acuña D., Cheng Y., Jiang X., Shi X. (2019). Printability of hydrogel composites using extrusion-based 3D printing and post-processing with calcium chloride. J. Food Sci. Nutr..

[50] Giuseppe M.D., Law N., Webb B.A., Macrae R., Liew L.J., Sercombe T.B., Dilley R.J., Doyle B.J. (2018). Mechanical behaviour of alginate-gelatin hydrogels for 3D bioprinting. J. Mech. Behav. Biomed. Mater..

[51] Mouser V.H., Melchels F.P., Visser J., Dhert W.J., Gawlitta D., Malda J. (2016). Yield stress determines bioprintability of hydrogels based on gelatin-methacryloyl and gellan gum for cartilage bioprinting. Biofabrication.

[52] Ma Y., Zhang C., Wang Y., Zhang L., Zhang J., Shi J., Si J., Yuan Y., Liu C. (2021). Direct three-dimensional printing of a highly customized freestanding hyperelastic bioscaffold for complex craniomaxillofacial reconstruction. Chem. Eng. J..

[53] Rahimnejad M., Labonte-Dupuis T., Demarquette N.R., Lerouge S. (2020). A rheological approach to assess the printability of thermosensitive chitosan-based biomaterial inks. Biomed. Mater..

[54] Lee J., Park S., Lee S., Kweon H.Y., Jo Y.-Y., Kim J., Chung J.H., Seonwoo H. (2023). Development of Silk Fibroin-Based Non-Crosslinking Thermosensitive Bioinks for 3D Bioprinting. Polymers.

[55] Elango J., Lijnev A., Zamora-Ledezma C., Alexis F., Wu W., Marín J.M.G., de Val E.M.S. (2023). The relationship of rheological properties and the performance of silk fibroin hydrogels in tissue engineering application. Proc. Biochem..

[56] Li V.C.-F., Dunn C.K., Zhang Z., Deng Y., Qi H.J. (2017). Direct Ink Write (DIW) 3D Printed Cellulose Nanocrystal Aerogel Structures. Sci. Rep..

[57] Habib M.A., Khoda B. (2022). Rheological analysis of bio-ink for 3D bio-printing processes. J. Manuf. Process..

[58] Habib A., Sathish V., Mallik S., Khoda B. (2018). 3D Printability of Alginate-Carboxymethyl Cellulose Hydrogel. Materials.

[59] Kaewpiroma S., Boonsang S. (2020). Influence of alcohol treatments on properties of silk-fibroin-based films for highly optically transparent coating applications. RSC Adv..

[60] Um I.C., Kweon H.Y., Lee K.G., Park Y.H. (2003). The role of formic acid in solution stability and crystallization of silk protein polymer. Int. J. Biol. Macromol..

[61] Qi P., Zeng J., Tong X., Shi J., Wang Y., Sui K. (2021). Bioinspired synthesis of fiber-shaped silk fibroin-ferric oxide nanohybrid for superior elimination of antimonite. J. Hazard. Mater..

[62] Daemi H., Barikani M., Barmar M. (2012). Synthesis and characterization of calcium alginate nanoparticles. Sci. Iran..

[63] Sharaf S., Hebeish A. (2015). Novel nanocomposite hydrogel for wound dressing and other medical applications. RSC Adv..

[64] Hidayat S., Ardiaksa P., Riveli N., Rahayu I. (2018). Synthesis and characterization of carboxymethyl cellulose (CMC) from salak-fruit seeds as anode binder for lithium-ion battery. J. Phys. Conf. Ser..

[65] Zhang H., Li L.-L., Dai F., Zhang H.-H., Ni B., Zhou W., Yang X., Wu Y.-Z. (2012). Preparation and characterization of silk fibroin as a biomaterial with potential for drug delivery. J. Transl. Med..

[66] Princem E., Kumachevam E. (2019). Design and applications of man-made biomimetic fibrillar hydrogels. Nat. Rev. Mater..

[67] Siqueira G., Kokkinis D., Libanori R., Hausmann M.K., Gladman A.S., Neels A., Tingaut P., Zimmermann T., Lewis J.A., Studart A.R. (2017). Cellulose Nanocrystal Inks for 3D Printing of Textured Cellular Architectures. Adv. Funct. Mater..

[68] Wang Y., Rudym D.D., Walsh A., Abrahamsen L., Kim H.J., Kim H.S., Kirker-Head C., Kaplan D.L. (2008). In Vivo Degradation of Three-Dimensional Silk Fibroin Scaffolds. Biomaterials.

